# Evaluating Premature Mortality in Bangladesh During the COVID‐19 Pandemic: A Comparative Analysis of Absolute and Relative Measures

**DOI:** 10.1002/puh2.70226

**Published:** 2026-04-09

**Authors:** Ahbab Mohammad Fazle Rabbi

**Affiliations:** ^1^ Department of Population Sciences University of Dhaka Dhaka Bangladesh

**Keywords:** Bangladesh, coronavirus disease 2019 (COVID‐19) pandemic, potential years of life lost (PYLL), premature mortality, threshold ages

## Abstract

The coronavirus disease 2019 (COVID‐19) pandemic significantly disrupted global mortality trends, affecting all age groups and leading to an increase in premature mortality. Accurately estimating premature mortality during such sudden epidemics is challenging, as different measures capture distinct aspects of mortality. Using period life tables from the 2024 World Population Prospects, this study assessed the impact of COVID‐19 on premature mortality in Bangladesh during 2020 and 2021 using four complementary measures: potential years of life lost (PYLL) to quantify the absolute burden of premature deaths, period‐expected years of life lost (PEYLL) to capture mortality exceeding expected levels, and two threshold ages based on lifespan disparity and life‐table entropy to reflect shifts in lifespan distribution and inequality. Results showed most measures of premature mortality in Bangladesh showed a temporary decline in 2020, followed by a partial rebound in 2021. For both sexes, PYLL and the crude PYLL rate decreased in 2020 relative to 2019 but increased again in 2021, reflecting a renewed rise in premature mortality during the second pandemic year. Relative measures exhibited more nuanced patterns: The lifespan‐disparity‐based threshold age declined for both males and females, whereas the entropy‐based threshold age decreased in 2020 for both sexes but slightly rebounded for males in 2021 and continued to decline for females, highlighting sex‐specific shifts in the distribution of deaths across ages. These findings demonstrate that applying multiple metrics provides a more nuanced understanding of how the pandemic affected different sexes and age groups, critical insights for guiding public health planning and pandemic response strategies.

## Graphical abstract

COVID‐19 temporarily disrupted Bangladesh's long‐term mortality decline, with increased levels of adult mortality during 2020 and 2021. Measures of premature mortality show a brief decline in 2020, followed by a rebound in 2021, underscoring the importance of monitoring age‐ and sex‐specific mortality patterns for public health planning and pandemic preparedness.

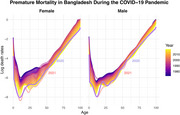

## 1 Introduction

Over the past few centuries, global human mortality has experienced a downward trend [1], a pattern that continued until the onset of 2020. The coronavirus disease 2019 (COVID‐19), caused by the SARS‐CoV‐2 virus, emerged as a worldwide pandemic at the onset of 2020 [2]. As of May 29, 2024, the World Health Organization (WHO) reported more than 775 million confirmed COVID‐19 cases globally, leading to 7 million deaths [3]. Mortality specific to COVID‐19 varied by both age and sex across different regions [4], with estimates of excess mortality differing across various phases of the pandemic [5]. The overall count of excess deaths attributed to COVID‐19 may be influenced by factors such as a population's age distribution and the healthcare infrastructure of the country. Clinical studies have consistently shown that the risk of death from COVID‐19 correlates with age [6]. The fatality rate of COVID‐19 increases progressively with age, and while both infants and older adults are at heightened risk for other respiratory illnesses [7], the focus has primarily been on the impact of excess mortality in older age groups compared to younger ones [8]. From a demographic viewpoint, the majority of severe cases affect older populations, particularly those aged 70 and above [9].

However, the pandemic has affected not only the elderly but also led to a global increase in mortality among younger populations, which is more likely to be classified as premature mortality [9, 10, 11]. The concept of premature mortality has become an important and widely utilized indicator for assessing the overall health of populations, as it provides valuable insights into the burden of avoidable deaths and health inequalities [8, 12]. Premature mortality refers to deaths that occur before a population‐specific threshold age, capturing deaths that occur earlier than expected relative to standard life expectancy [12, 13]. An increasing trend in premature deaths may serve as an indicator for policymakers of a potential underlying health issue within the population. Evaluating premature mortality is crucial for understanding both global and national levels of unnecessary or preventable mortality burdens [14]. Furthermore, premature mortality is closely linked to health disparities, meaning that reducing early mortality can lead to longer lifespans and greater equality among individuals [14].

Building on the concept of premature mortality, quantification methods generally fall into two categories: absolute and relative measures. Absolute measures use fixed age thresholds to define premature deaths, whereas relative measures consider the age distribution at death [14]. Among the most widely used indicators is potential years of life lost (PYLL), an absolute measure based on various definitions of thresholds [15]. For instance, the OECD defines premature mortality as death before age 70 [12], whereas the WHO and Eurostat apply alternative criteria [14]. Although not strictly a measure of premature mortality, population‐expected years of life lost (PEYLL) is also used to quantify the burden of mortality by comparing age at death to population‐specific life expectancy. Relative measures, on the other hand, avoid the arbitrariness of thresholds and better accommodate differences in mortality patterns across populations; by using the shape of the age‐at‐death distribution (Lexis 1879), they allow for more comparable assessments across countries with varying life expectancy [14]. Different measurement approaches can lead to varying conclusions about the extent of premature mortality and the relative success of populations in addressing it. Relying solely on either absolute or relative measures provides an incomplete picture of premature mortality dynamics. Absolute indicators like PYLL and PEYLL reveal the scale of life lost due to early deaths, but they do not capture how mortality patterns shift across age groups [14]. Relative measures, such as threshold ages based on lifespan disparity and life‐table entropy, complement this by identifying changes in the distribution and timing of deaths [16]. For example, an increase in PYLL combined with a decline in threshold ages suggests not just a rise in early deaths, but also a compression in lifespan [17].

Since the onset of the COVID‐19 pandemic, numerous studies have examined changes in premature mortality. However, the majority of these investigations have focused on low‐mortality countries with aging populations, primarily due to the availability of reliable and high‐quality mortality data (see, e.g., [8]). Most of these studies rely on classical absolute measures of premature mortality, which vary across contexts and stages of the pandemic [18]. Yet, a substantial gap persists in the literature concerning the impact of excess mortality in high‐mortality countries—particularly those undergoing mortality transition. Populations in these contexts are typically younger compared to aging societies, and as a result, the use of absolute measures—often based on fixed benchmark ages—may provide a misleading profile of premature mortality. Although some multi‐country analyses have assessed life years lost due to the pandemic, they often neglect relative measures that account for the distribution of deaths across the entire lifespan [19]. Sole reliance on absolute indicators thus risks oversimplifying the true burden of premature mortality, particularly in younger populations where age‐at‐death distribution plays a critical role in interpretation [16, 20].

This gap in the literature, particularly the limited focus on high‐mortality countries undergoing demographic transition, is especially relevant for countries like Bangladesh. As a lower middle‐income country undergoing a demographic and mortality transition, Bangladesh has experienced steady gains in life expectancy in recent decades, driven by falling mortality and near‐replacement fertility rates [21]. However, its population remains relatively young, and these demographic shifts have not yet translated into the age structures typical of high‐income, aging societies [4]. During the COVID‐19 pandemic, although Bangladesh reported a lower overall mortality burden than some neighboring countries [22], a significant share of excess deaths occurred among younger age groups, highlighting the limitations of using fixed‐age absolute measures alone to capture the true impact. This article aims to examine the changes in premature mortality levels in Bangladesh during the COVID‐19 pandemic. Besides using PYLL to quantify the absolute burden, we apply two age‐threshold‐based relative measures to assess changes in premature mortality during the pandemic. These relative measures not only provide a more dynamic understanding of premature mortality but also reflect how mortality patterns shifted during the pandemic in a context of demographic transition. Rather than seeking a single “best” definition of premature mortality, this study analyzes both absolute and relative measures to provide a comprehensive assessment of the pandemic's impact. Moreover, we compared the premature mortality patterns in Bangladesh with four neighboring South Asian countries to contextualize the findings regionally. By integrating these complementary metrics, we aim to show how different conceptualizations of premature mortality jointly illuminate the pandemic's demographic toll in Bangladesh—a younger, high‐mortality population undergoing rapid mortality transition.

## 2 Data and Methods

The following subsection provides details on data used in this study and different methods to measure premature mortality.

### 2.1 Data

Due to lack of detailed mortality data from vital registration systems, we considered alternative data sources for the current study. The data used in this study are derived from the 2024 revision of the World Population Prospects (WPP 2024). For Bangladesh, WPP 2024 provides estimated life tables in single years, which are constructed up to age 100 years. However, due to the presence of zero mortality rates in the upper ages for most of the years for Bangladesh, we reconstructed these life tables up to age 85 years for our analysis. Detailed information regarding the data sources and estimation procedures is discussed elsewhere. Since Bangladesh became independent at the end of 1971, we have considered data from 1972 to 2021 for the current study. The long historical span provides context for the exceptional disruption of 2020–2021 by situating the pandemic against nearly five decades of declining premature mortality in Bangladesh.

### 2.2 Methods

Standard life‐table notations are used all over the article. Age‐specific mortality rates for a period life table are defined as

m(x)=D(x)/P(x);

where *D*(*x*) is the observed death counts in a calendar year, and *P*(*x*) is the mid‐year population of that year for age group *x*.

#### 2.2.1 Absolute Measures of Premature Mortality

##### 2.2.1.1 Period Expected Years of Life Lost

We estimated the PEYLL to measure the overall change in mortality during pandemic rather than the premature mortality only. The PEYLL demonstrates the mortality gap between the current period of life expectancy at a given age and the actual age at the time of death. Symbolically,

(1) $$\mathrm{PEYLL}=\sum_{x=0}^{\omega }d\left(x\right)\cdot e\left(x\right),$$

where *d*(*x*) is the life‐table distribution of death at each age *x, e*(*x*) is the remaining life expectancy at age *x*, and *ω* is the maximum attainable age in the life table.

##### 2.2.1.2 Potential Years of Life Lost

We considered the PYLL as the main indicator of premature deaths [15]. PYLL provides more conservative estimates than other indicators, and it focuses on the premature mortality of those who die [8]. Symbolically,

(2) $$\mathrm{PYLL}=\sum_{x=0}^{\;\tau }\left(\tau -x\right)\cdot \;d\left(x\right),$$

where *τ* is the potential limit of life (cut‐off point in lifespan). Following OECD (2009), we considered 70 years as the cut‐off age for premature mortality. This choice of upper limit (70 years) ensures that deaths happening over 70 years of age contribute zero PYLL to the calculation. Another benefit of this methodology is that it also assumes uniform distribution of deaths within the age groups [8, 15]. PYLL are calculated per person death and as rates (per 100,000 population) using the following equation:

(3) $$\begin{array}{ccc} & & \mathrm{Crude}\;\mathrm{PYLL}\;\mathrm{rate}\\ & = & \left(\mathrm{PYLL}/\mathrm{Population}\;\mathrm{under}\;\mathrm{age}\;70\;\mathrm{years}\right)\times 100,000. \end{array}$$

#### 2.2.2 Relative Measures of Premature Mortality

Two different definitions of threshold ages are considered in this study. Both of them are defined as a specific age that divides premature deaths from the late deaths.

##### 2.2.2.1 Threshold Age Based on Lifespan Disparity

The first measure of the threshold age was proposed by Zhang and Vaupel [23], and this measure is based on lifespan disparity. To measure lifespan disparity, we utilize the definition of Vaupel and Romo [20] and Zhang and Vaupel [23] where it is defined as the average number of life years lost at birth (as a result of death). Symbolically,

(4) $$e_{0^{†}}=\frac{\int_{0}^{\omega e}\left(x\right)d\left(x\right)dx}{l\left(0\right)},$$

where *l(x)* is the number of people alive at age *x* (*l*0 is the life‐table radix). It should be noted that, despite several other alternative formulations of *e* ^†^ (see [20], for an example), the difference between these measures are negligible in terms of outcomes and sensitivity analysis [16]. Hence, the threshold age proposed by Zhang and Vaupel [23] is denoted by $a^{†}$ is the age at which,

(5) $$e^{†}=e\left(a\right)\left(1-H\left(a\right)\right),$$

where *e*(*a*) is the life expectancy for threshold age *a* and $H\left(a\right)=\int_{0}^{a}\mu \left(x\right)dx$ is the cumulative hazard to age *a*. This estimation is based on the assumption that the life‐table entropy (defined in the next subsection) should be less than one. $a^{†}$ can be estimated using interpolation routines in most of the widely used statistical software.

##### 2.2.2.2 Threshold Age Based on Life Table Entropy

The second measure of threshold age between premature deaths and late deaths is based on life‐table entropy [17]. Life‐table entropy is defined as a measure of elasticity of life expectancy to a change in mortality [17]. Symbolically,

(6) $$H=\frac{\int_{0}^{\omega }l\left(a\right)\ln\left(l\left(a\right)\right)da}{\int_{0}^{\omega }l\left(a\right)da}=\int_{0}^{\omega }c\left(a\right)H\left(a\right)da=\frac{e_{0}^{†}}{e\left(0\right)},$$

where *c(a)* is the population age structure; *H(a)* is the cumulative hazard to age *a*. Later, Aburto et al. [17] defined a measure of threshold age between premature deaths and later deaths as a relative change in H. To compute this measure, Aburto et al. [17] defined entropy of life table at age *x* as a conditional measure (on surviving to age *x*). Symbolically,

(7) $$\bar{H}\;\left(x\right)=\;\frac{\int_{x}^{\omega }l\left(a\right)\ln\left(l\left(a\right)\right)da}{\int_{x}^{\omega }l\left(a\right)da}=\frac{e_{x}^{†}}{e\left(x\right)}.$$

Assuming an improvement in mortality over all ages, the threshold age defined by Aburto et al. [17] separates positive from negative contributions to H resulting from mortality improvements. Since mortality improvement over time occurs at all ages, there exists a unique threshold age $a^{H}$ that separates positive from negative contributions to the life‐table entropy H. This threshold age $a^{H}$ is reached when

(8) $$H\left(a^{H}\right)+\bar{H}\left(a^{H}\right)=1+H.$$

#### 2.2.3 Ethical Considerations

We used estimated life tables of the United Nations for this study. These life tables do not contain any individual's identifiable information, rather they are aggregate measures. In addition, no human subject was directly involved in the present study. Hence, this study was exempt from any ethical or Institutional Review Board clearance.

## 3 Results

### 3.1 Ongoing Mortality Transition and COVID‐19 Pandemic in Bangladesh

**FIGURE 1:**
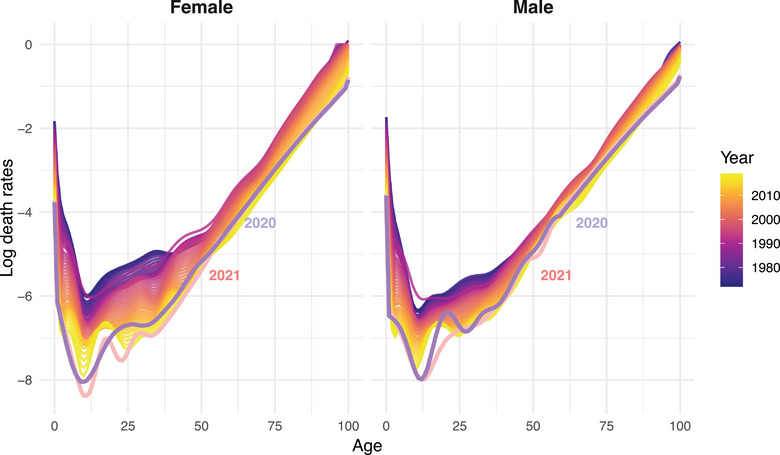
Age‐ and sex‐specific mortalit of Bangladesh for the period 1972–2021 (WPP 2024).

Figure 1 presents age‐ and sex‐specific log death rates for Bangladesh based on WPP 2024 estimates from 1972 to 2021. Using a log scale makes it possible to visualize mortality changes consistently across the life course and over a long historical period. Two temporary mortality disruptions are visible in the early part of the series. The first corresponds to the 1974–1975 flood‐induced famine, which produced a notable rise in mortality. A second disturbance around 1991 aligns with the severe cyclone that caused significant mortality, particularly in coastal areas. Pandemic years (2020 and 2021) are highlighted in Figure 1 to emphasize recent shifts relative to the pre‐pandemic trend. The figure shows a continued mortality decline over the decades, but a reversal during the pandemic years. In 2020 and especially in 2021, mortality rates increased, most visibly at younger and middle ages (below age 60) and more sharply for males. This excess mortality at working and reproductive ages is particularly relevant because it directly contributes to premature mortality, as captured by the absolute (PEYLL, PYLL) and relative ($a^{†}$, $a^{H}$) measures analyzed in the following sections.

### 3.2 Absolute Measures of Premature Mortality

In this subsection, we present the findings from the absolute measures of premature mortality. All results are reported separately for males and females throughout this section to facilitate sex‐specific comparisons. Before focusing specifically on premature mortality, we first examined the PEYLL to capture the overall mortality burden during the pandemic period. PEYLL decreased in 2020 for both sexes but increased again in 2021. Among Bangladeshi males, PEYLL declined until 2020 and then rose slightly in 2021. In 2019, the PEYLL for Bangladeshi males was 14.16 years. This measure declined to 13.56 years in 2020 but increased to 14.14 years in 2021. The detailed trend of PEYLL is presented in Figure S1. The PYLL showed a similar pattern in Bangladesh, with a decline in 2020, followed by an increase in 2021. In 2019, the PYLL for Bangladeshi females was 5.80 years. This value decreased slightly to 5.61 years in 2020 but then rose to 6.07 years in 2021. However, males exhibited higher PYLL values throughout the study period.

**FIGURE 2:**
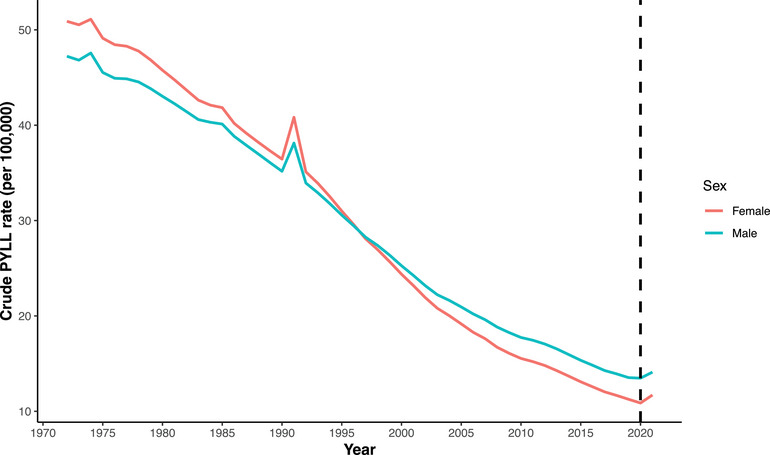
Trends in the crude potential years of life lost (PYLL) rate in Bangladesh by sex, 1972–2021 (per 100,000 population).

Figure 2 illustrates the crude PYLL rate, with 2020, the first year of the pandemic, highlighted using a bold dashed line. The crude PYLL rate showed a pattern similar to the other absolute measures. At the beginning of the period analyzed, the crude PYLL rate was higher for both sexes in Bangladesh but declined in subsequent years. In 2019, the crude PYLL rate was 13.53 per 100,000 population for males and 11.26 per 100,000 population for females. For Bangladeshi females, this declining trend continued in 2020 (10.88), followed by a slight increase in 2021 (11.71). A similar pattern was observed for males.

### 3.3 Relative Measures of Premature Mortality

**FIGURE 3:**
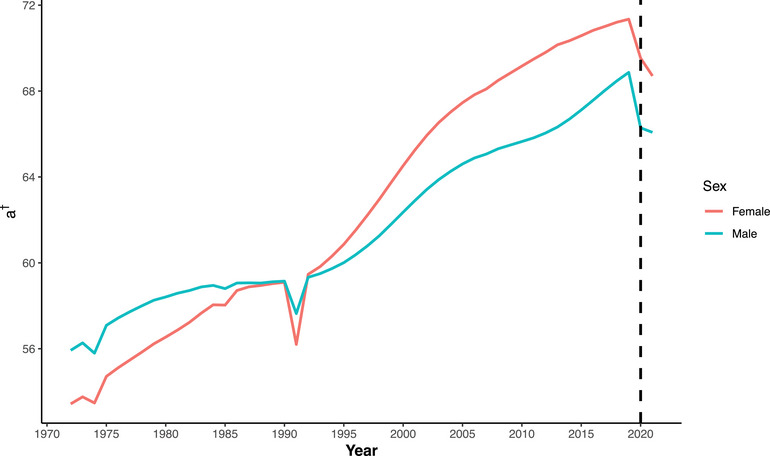
Trend of threshold age based on lifespan disparity ($a^{†}$) in Bangladesh for both sexes (1972:2021).

**FIGURE 4:**
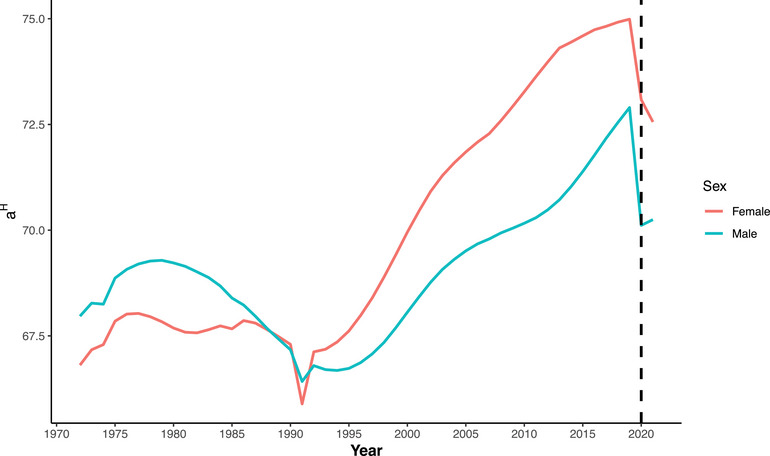
Trend of threshold age based on life‐table entropy ($a^{H}$) in Bangladesh for both sexes (1972:2021).

This subsection presents the findings from the relative measures of premature mortality. We calculated two different threshold ages as relative measures of premature mortality. These measures are illustrated in Figures 3 and 4. The first measure is based on lifespan disparity, denoted as $a^{†}$. This measure assesses the variability in age at death across a population, providing insights into how inequality in lifespan contributes to premature mortality. The second measure is based on life‐table entropy, denoted as $a^{H}$. Life‐table entropy captures the uncertainty or dispersion in the distribution of age at death, offering another perspective on lifespan inequality. The detailed estimation procedures and calculations for these relative measures are provided in Figures S2–S4. These calculations include the methods for determining the threshold ages and interpreting the results within the context of premature mortality. Figures 3 and 4 visually represent the trends and changes in these relative measures over time, providing a comprehensive view of how premature mortality has evolved in relation to lifespan disparity and life‐table entropy.

The threshold age based on lifespan disparity ($a^{†}$) showed a decreasing trend for both sexes, continuing the decline observed since 2019. However, the rate of decline slowed in 2021 for both sexes, even though mortality was higher in that year. In 2019, $a^{†}$ was 68.87 years for males which decreased to 66.29 years in 2020 and 66.08 years in 2021. A slightly different pattern was observed for the entropy‐based threshold age. The entropy‐based threshold age ($a^{H}$) decreased in 2020 for males, followed by a slight increase in 2021. However, for females, it continued to decline throughout the pandemic period. The value of $a^{H}$ for males declined from 72.90 years in 2019 to 70.11 years in 2020, followed by a slight increase to 70.25 years in 2021. Among Bangladeshi females, both threshold ages were consistently higher than those of males but also declined during the pandemic period. The lifespan‐disparity‐based threshold age ($a^{†}$) decreased from 71.34 years in 2019 to 69.53 years in 2020 and further to 68.71 years in 2021. Similarly, the entropy‐based threshold age ($a^{H}$) declined from 74.98 years in 2019 to 73.09 years in 2020 and then to 72.56 years in 2021. A summary of changes in all these measures (both absolute and relative) and life expectancy at birth (*e*(0)) for the years 2019 to 2021 is provided in Table 1.

**TABLE 1:** Changes in life expectancy and measures of premature mortality in Bangladesh during pandemic (2019:2021).

	Years	*e*(0)	PEYLL	PYLL	Crude PYLL rate (per 100,000)	$a^{†}$	$a^{H}$
Male	2019	70.93	14.16	7.13	13.53	68.87	72.90
	2020	69.58	13.56	7.11	13.47	66.29	70.11
	2021	69.48	14.14	7.47	14.11	66.08	70.25
Female	2019	74.40	13.73	5.80	11.26	71.34	74.98
	2020	73.43	13.30	5.61	10.88	69.53	73.09
	2021	72.84	13.81	6.07	11.71	68.71	72.56

*Note:* e(0) stands for life expectancy at birth; PEYLL stands for period expected years of life lost; PYLL stands for potential years of life lost; $a^{†}$ is the threshold age derived from lifespan disparity; and $a^{H}$ is the threshold age based on life table entropy.

### 3.4 Comparison With Other South Asian Countries

**FIGURE 5:**
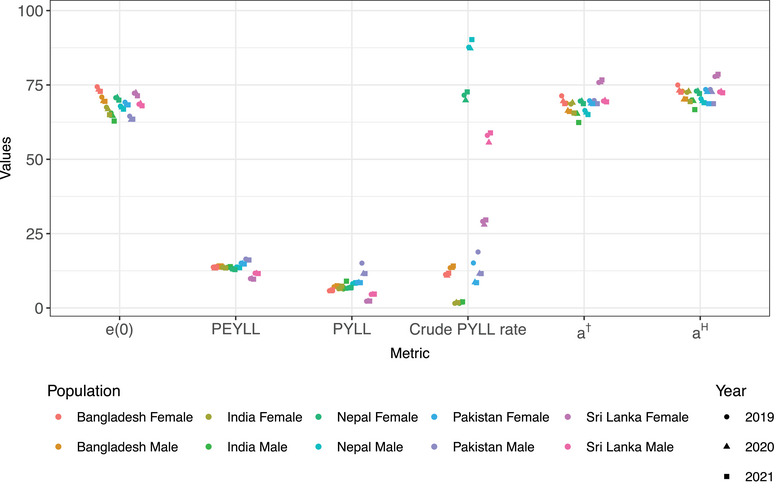
Trend of threshold age based on life‐table entropy ($a^{H}$) in Bangladesh for both sexes (1972:2021).

Life expectancy at birth and selected measures of premature mortality for Bangladesh and four neighboring countries are compared in Figure 5, where each population (country‐sex combination) is shown in a distinct color for ease of comparison and years are plotted with different shapes. Across the premature mortality indicators, Bangladesh generally follows patterns similar to several neighboring populations, although notable deviations exist. For PEYLL, both males and females in Bangladesh show levels comparable to India and Nepal, whereas Sri Lanka exhibits consistently lower values and Pakistan higher values, indicating that Bangladesh broadly aligns with the regional middle range for this measure. For PYLL, Bangladeshi males show values similar to India, whereas Nepal and Pakistan display higher levels and Sri Lanka markedly lower ones. Among females, Bangladesh lies near the regional midpoint, with Sri Lanka showing the lowest values and Pakistan the highest, suggesting partial similarity but clear cross‐country variation. The crude PYLL rate differs substantially from Bangladesh for most populations. Both Nepal and Sri Lanka display markedly higher rates for males and females, whereas India shows extremely low values. Pakistan remains somewhat closer but still deviates, indicating that Bangladesh does not closely mirror other populations for this rate‐based measure.

However, for the relative threshold measures, patterns are more consistent (Figure 5). Values of $a^{†}$ in Bangladesh are broadly comparable to India and Nepal, whereas Sri Lanka tends to exhibit higher ages, particularly for females. Pakistan remains close to Bangladesh for males but slightly higher for females. Similarly, $a^{H}$ shows broadly parallel patterns across countries, with Bangladesh generally falling between India (lower) and Sri Lanka (higher), and Nepal and Pakistan displaying similar intermediate levels.

## 4 Discussion

Efforts to quantify premature mortality remain a central concern in epidemiology and demographic research, particularly during periods of sudden mortality shocks such as the COVID‐19 pandemic. Using multiple indicators, including PYLL, threshold ages derived from lifespan disparity ($a^{†}$) and life‐table entropy ($a^{H}$), and PEYLL, this study examined how the pandemic affected mortality patterns in Bangladesh. Most measures showed a temporary decline in 2020, followed by a partial increase in 2021, reflecting the evolving mortality impact of the pandemic. For both males and females, PYLL and the crude PYLL rate decreased in 2020 relative to 2019 but increased again in 2021, indicating a renewed rise in premature mortality during the second pandemic year. The relative measures showed more nuanced patterns. The threshold age based on lifespan disparity ($a^{†}$) declined during the pandemic period for both sexes, suggesting that a larger share of deaths occurred at ages considered premature relative to the evolving mortality schedule. In contrast, the entropy‐based threshold age ($a^{H}$) declined in 2020 for both sexes but showed a slight rebound for males in 2021 while continuing to decrease for females, indicating sex‐specific differences in how mortality changes were distributed across ages. Taken together, these results suggest that although the pandemic temporarily disrupted the long‐term mortality transition in Bangladesh, its effects on premature mortality were not uniform across measures or between sexes. Absolute measures capture the overall burden of premature deaths, whereas the relative threshold measures reflect changes in the distribution of deaths across the life course. PEYLL, by contrast, reflects the broader mortality burden and should not be interpreted strictly as a measure of premature mortality.

Our estimated trend of PYLL showed a decline in 2020, followed by an increase in 2021 for both males and females. This pattern reflects the higher mortality levels observed in 2021 compared to the first year of the pandemic. The rise in deaths across both sexes in 2021 coincided with the spread of the Delta variant, which drove the second and most severe wave of the pandemic in South Asia [24]. Because PYLL incorporates both the number of deaths and the age at which they occur, it is particularly sensitive to changes in premature mortality [15]. The increase in PYLL in 2021 therefore indicates a renewed rise in premature mortality following the temporary decline observed in 2020.

Unlike standard PYLL, the crude PYLL rate accounts for the population under age 70 when summarizing premature mortality. In Bangladesh, the crude PYLL rate followed a pattern similar to PYLL, declining in 2020 and increasing again in 2021 for both sexes. Comparable increases during the pandemic have been reported in other countries when compared with pre‐pandemic trends [8]. However, the magnitude of change in the crude PYLL rate was smaller than the change observed in PYLL for both sexes. Differences between our findings and those of other studies may partly reflect methodological choices, particularly the definition of the upper age threshold for premature mortality. Although we adopted the widely used cut‐off age of 70 years [12], other studies have used life expectancy at birth as the reference threshold [18]. As noted by Mazzuco et al. [14], such absolute measures require the selection of a specific threshold, which can influence estimates of premature mortality. However, the crude PYLL rate is strongly influenced by the underlying population structure. This effect explains why Indian males and females show very low crude PYLL rates compared to Bangladesh and other neighboring countries, despite comparable absolute mortality levels (Figure 5).

The combination of absolute (PYLL) and relative ($a^{†}$, $a^{H}$) measures clarifies how fixed cutoffs compare with distribution‐sensitive thresholds in identifying the age groups most affected by premature deaths. Relative measures help capture differences in how certain groups or sectors are affected, particularly when population characteristics (e.g., age, sex) play a significant role. In the case of the COVID‐19 pandemic, relative measures capture shifts in the age distribution of deaths and provide a clearer picture of how mortality shifts across various populations during such an unprecedented event. This approach aligns with understanding population‐based impacts, revealing hidden vulnerabilities and informing targeted interventions. Thus, in relative measures of premature mortality, the share of premature deaths depends on the entire distribution of deaths by age [14]. Unlike crude PYLL rate, relative measures are less sensitive to population size and thus show more consistent patterns across countries (Figure 5). These measures provide a better comparison of premature mortality patterns across South Asian populations. The exact operationalization of premature mortality also depends on the pattern of senescent mortality, and each country has its own definition of old age mortality. In this regard, we used two different threshold ages based on lifespan disparity and life‐table entropy. These measures provide complementary perspectives on the distribution of deaths by age [17, 23]. Interpreting $a^{†}$ and $a^{H}$ together helps illustrate how mortality changes during the pandemic shifted the boundary between premature and later deaths, and how these shifts differed between males and females.

The threshold ages based on lifespan disparity ($a^{†}$) proved to be more sensitive to changes in mortality over time, particularly during the pandemic when excess mortality occurred in Bangladesh, compared to absolute measures. This sensitivity aligns with the findings from Aburto et al. [17], where the threshold age based on life‐table entropy was shown to distinguish between the positive and negative contributions of mortality reductions. In particular, it indicates that reductions in mortality below certain age thresholds improve life expectancy, whereas those above contribute to lifespan inequality. Unlike absolute measures of premature mortality, $a^{†}$ showed a consistent decreasing trend for both sexes. In contrast, $a^{H}$ showed a decreasing trend for Bangladeshi females during the pandemic years, a pattern consistent with the dynamic nature of relative measures. For Bangladeshi males, $a^{H}$ decreased in 2020 and then increased in 2021, reflecting the shifts in mortality during the pandemic. These patterns help illustrate how the boundary between premature and later deaths shifted during the pandemic and how these shifts differed between males and females.

The primary aim of developing both threshold ages was to identify an age in the human lifespan that hypothetically separates the positive and negative effects of age‐specific mortality improvements [17]. Empirical studies from low‐mortality countries have shown that $a^{†}$ typically falls below the life expectancy at birth [16]. Additionally, Aburto et al. [17] found that $a^{H}$ tends to be higher than $a^{†}$, which was consistent with our findings. We also obtained similar trend for Bangladesh. Relative measures of premature mortality, such as these threshold ages, recognize that the boundary between premature and later deaths depends on the underlying mortality schedule and may therefore vary across populations and over time [14]. Comparison with neighboring South Asian countries indicates that Bangladesh had almost similar trend for most premature mortality indicators. Absolute measures, particularly the crude PYLL rate, differ substantially due to population‐size effects, resulting in very low values for Indian males and females. In contrast, relative measures ($a^{†}$ and $a^{H}$) are broadly consistent across countries, showing that Bangladesh experienced similar patterns of premature mortality during the COVID‐19 pandemic compared to India, Nepal, and Pakistan, with Sri Lanka generally exhibiting higher threshold ages. These observations suggest that while Bangladesh was not an outlier in the region, the pandemic's impact on premature mortality had sex‐ and country‐specific nuances that align with population structure and underlying mortality schedules.

These findings have important implications for public health planning and policy. By using both absolute (PYLL) and relative ($a^{†}$, $a^{H}$) measures, policymakers can identify not only the overall burden of premature deaths but also which age groups and sexes are most vulnerable. Such insights can inform targeted interventions, resource allocation, and pandemic preparedness strategies. Moreover, understanding the shifts in lifespan inequality provides guidance on addressing systemic vulnerabilities in health systems, ensuring that future public health responses are more equitable and effective in protecting high‐risk populations.

Although this study provides valuable demographic insights into shifts in premature mortality during the COVID‐19 pandemic in Bangladesh, it is important to recognize its limitations regarding causal inference. The analysis relies on aggregate life‐table data and does not incorporate individual‐level information on underlying health conditions, socio‐economic status, or other structural factors known to influence mortality risk. Consequently, the observed gender and age patterns in premature mortality may reflect a complex interplay of COVID‐19 exposure, comorbidities, and socio‐economic determinants. This study does not disentangle these factors nor attribute excess mortality solely to the pandemic. Future research incorporating detailed individual‐level data and multivariate analyses will be essential to understand the causal mechanisms and inform targeted public health interventions. Nonetheless, our demographic analysis offers an important population‐level overview of mortality changes during this period, providing a foundation for further investigation.

### 4.1 Strength and Limitations

Our study is one of the few studies that attempt to evaluate the burden of COVID‐19 on premature mortality using both absolute and relative approaches. Additionally, this study explored relative measures of premature mortality by considering threshold ages based on demographic measures of lifespan variation, rather than relying solely on statistical measurements. To the best of our knowledge, this study is the first to utilize such threshold ages as relative measures of premature mortality and apply them to assess the impact of excess mortality caused by the COVID‐19 pandemic. Nationally representative data improve reliability compared to regional or publicly available datasets typically used in high‐mortality countries.

The analysis relies on smoothed national mortality estimates from the 2024 revision of WPP revision [4], which may partially obscure year‐to‐year variability in actual mortality. The study focuses on overall trends rather than detailed age‐specific contributions to premature mortality, which have been extensively reported in prior studies [8]. Finally, the use of OECD cut‐off ages ensures comparability with other countries but may not fully reflect Bangladesh‐specific life expectancy patterns; using national life expectancy could refine estimates at the cost of comparability.

## 5 Conclusion

This article presents a comprehensive analysis of changes in premature mortality in Bangladesh during the COVID‐19 pandemic, quantifying its effects on age‐ and sex‐specific mortality through two types of measures. The results indicated a decrease in premature mortality for Bangladeshi males in 2020, reflecting the continuation of the ongoing mortality transition, followed by an increase in 2021. In contrast, increased premature mortality was observed for Bangladeshi females in both years. These patterns, observed through absolute (PYLL) and relative ($a^{†}$, $a^{H}$) measures, highlight differences in how the pandemic affected age groups and sexes, whereas PEYLL underscores excess mortality relative to expectations. The findings demonstrate that absolute measures capture the overall burden of premature deaths, whereas relative measures provide insights into changes in the distribution of deaths and lifespan inequality. For example, males experienced a temporary decline in PYLL and *a* ^†^ in 2021, whereas females showed consistent increases in PYLL, reflecting the differential impact of COVID‐19 by sex. Relative threshold ages ($a^{†}$, $a^{H}$) further identify the age groups most affected, offering a nuanced view of how mortality patterns shifted during the pandemic.

A significant challenge in this field is that premature mortality is an inherently abstract concept, making precise estimation difficult. The way the concept is operationalized can significantly influence the results. It is common for studies on premature mortality to show that one population might appear to have a more favorable outcome compared to another based on one measure, but worse outcomes based on a different measure. Additionally, the population under study may still be undergoing mortality transition or be in a high‐mortality regime, which makes such comparisons inherently problematic.

Insights into the impact of the COVID‐19 pandemic on premature mortality in Bangladesh have been offered by this study, though opportunities for further exploration remain. A more detailed analysis incorporating age‐specific decomposition and cause‐specific mortality could provide a clearer picture of the pandemic's effects. The use of WPP data was due to the lack of comprehensive registration systems in Bangladesh, but applying these methods to nationally representative data could offer more insights. Additionally, comparing premature mortality during the pandemic with neighboring countries might have clarified the observed relative changes in mortality. Combining features of both approaches could enhance the comparability of estimates across similar populations. Addressing these aspects would deepen our understanding of the pandemic's impact on premature mortality and longevity in Bangladesh, potentially guiding more effective public health policies and interventions.

## Author Contributions

**Ahbab Mohammad Fazle Rabbi**: conceptualization, methodology, validation, visualization, writing – review and editing, software, formal analysis, data curation, writing – original draft.

## Funding

The author has nothing to report.

## Conflicts of Interest

The author declares no conflicts of interest.

## Transparency Statement

The corresponding author confirms that this manuscript is an honest, accurate, and transparent account of the study being reported; that no important aspects of the study have been omitted; and that any discrepancies from the study as planned (and, if relevant, registered) have been explained.

## Supporting information




**Supporting File 1**: puh270226‐sup‐0001‐SuppMat.pdf

## Data Availability

The data used in this study are free and available for access in World Population Prospects.
