# Correction to “Evaluating Premature Mortality in Bangladesh During the COVID‐19 Pandemic: A Comparative Analysis of Absolute and Relative Measures”

**DOI:** 10.1002/puh2.70276

**Published:** 2026-05-18

A. M. F. Rabbi, “Evaluating Premature Mortality in Bangladesh During the COVID‐19 Pandemic: A Comparative Analysis of Absolute and Relative Measures.” *Public Health Challenges* 5, no. 2 (2026): e70226, https://doi.org/10.1002/puh2.70226.

**1**. In Section 2.2.2.1, Equation (4) and its accompanying statement as well as Equation (7) were published incorrectly. Equation **(4)** and its statement should display as follows: Symbolically,

$$\;e_{0}^{†}=\frac{\int_{0}^{\omega }e\left(x\right)d\left(x\right)dx}{l\left(0\right)}.$$

where *l*(*x*) is the number of people alive at age *x* (*l*(0) is the life‐table radix).

**2**. **Equation (7)** should display as follows:

$$\bar{H}\left(x\right)=\;\;\frac{\int_{x}^{\omega }l\left(a\right)\ln\left(l\left(a\right)\right)da}{\int_{x}^{\omega }l\left(a\right)da}=\frac{e_{x}^{†}}{e\left(x\right)}.$$

**3**. Both Figures 1 and 5 captions were published incorrectly.

**Figure 1** caption should read as follows: Age‐ and sex‐specific mortality of Bangladesh for the period 19722021 (WPP 2024).

**4**. **Figure 5** caption should read as follows: “Changes in life expectancy at birth and premature mortality in Bangladesh and selected neighboring countries during 2019–2021.”

We apologize for these errors.

