# Supplementary material for: Evaluating Premature Mortality in Bangladesh During the COVID‐19 Pandemic: A Comparative Analysis of Absolute and Relative Measures
Source: Public Health Chall. 2026 Apr 9;5(2):e70226. doi: 10.1002/puh2.70226 (PMC13064581; doi:10.1002/puh2.70226)
Supplement: Supplementary file 1 — Supporting File 1: puh270226‐sup‐0001‐SuppMat.pdf [file PUH2-5-e70226-s001.pdf]

Evaluating Premature Mortality in Bangladesh During the COVID-19 Pandemic: A Comparative Analysis of Absolute and Relative Measures

Ahbab Mohammad Fazle Rabbi*

Department of Population Sciences, University of Dhaka, Dhaka, Bangladesh

Supplementary materials

Trend of PEYLL in Bangladesh

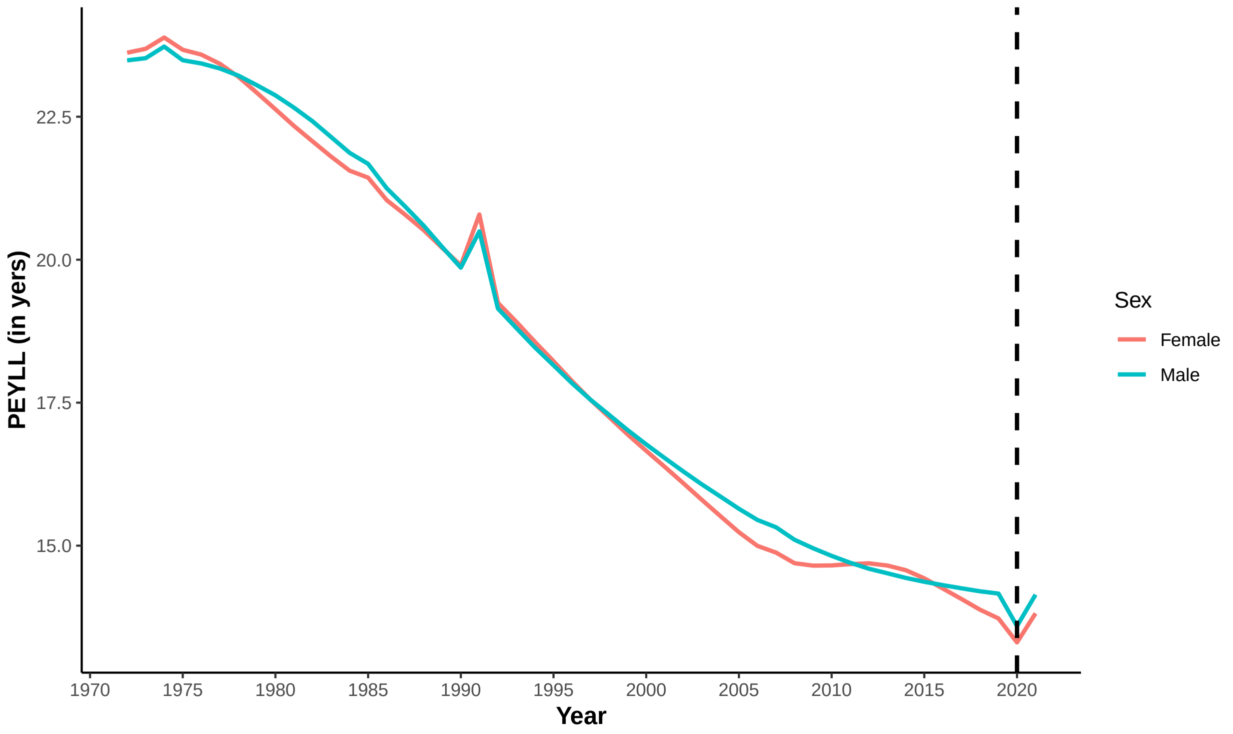

Figure S1: Trend of potential expected years of life lost (PEYLL) in Bangladesh for both sexes (1972:2021).

*Corresponding: fazlerabbi@du.ac.bd

Illustration of the estimation procedure of different threshold ages for Bangladesh

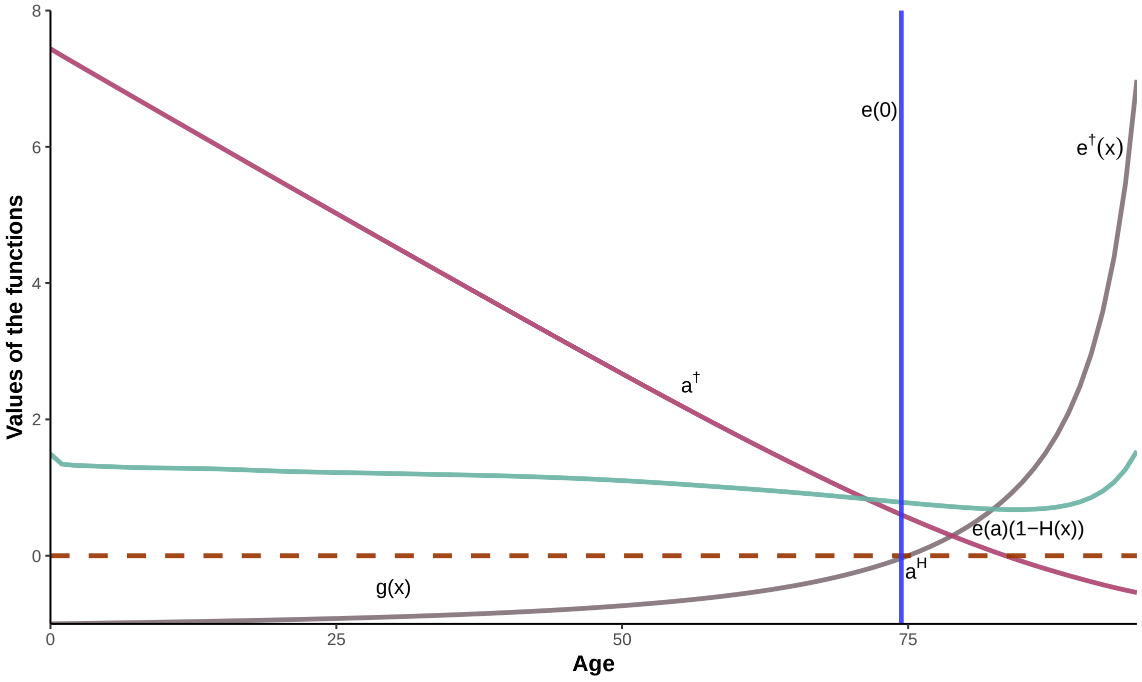

Figure S2: Estimation procedure of different threshold ages for Bangladeshi females in the year 2019 as an example.

Trend of lifespan disparity in Bangladesh

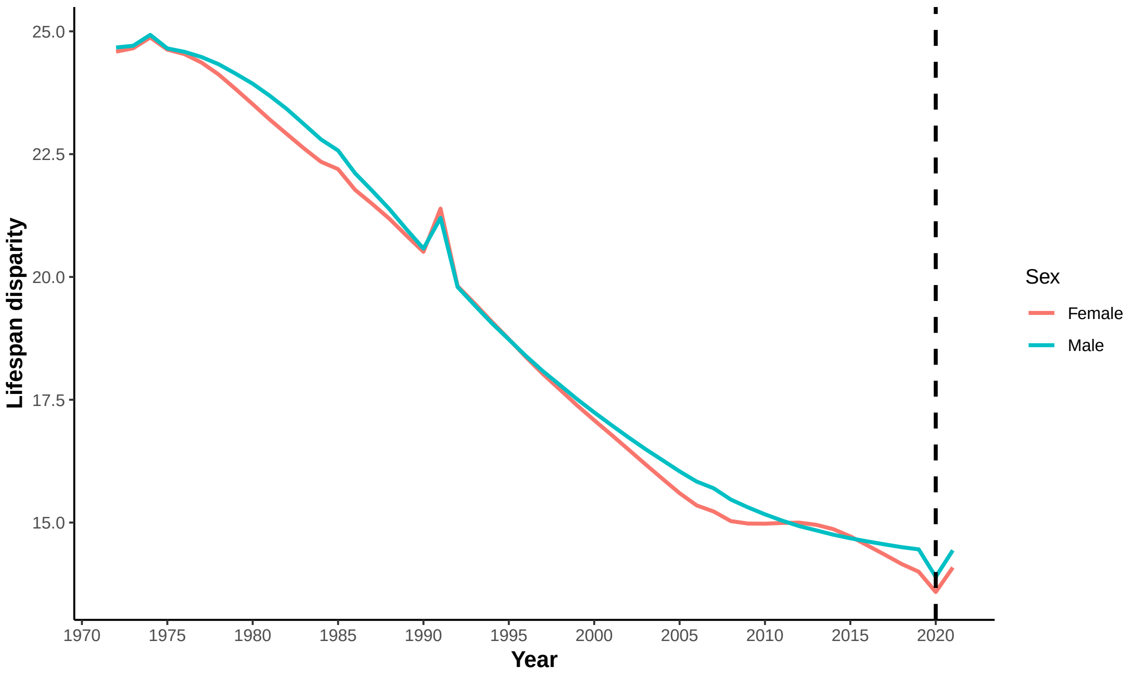

Figure S3: Trend of lifespan disparity in Bangladesh for both sexes (1972:2021).

Trend of life table entropy in Bangladesh

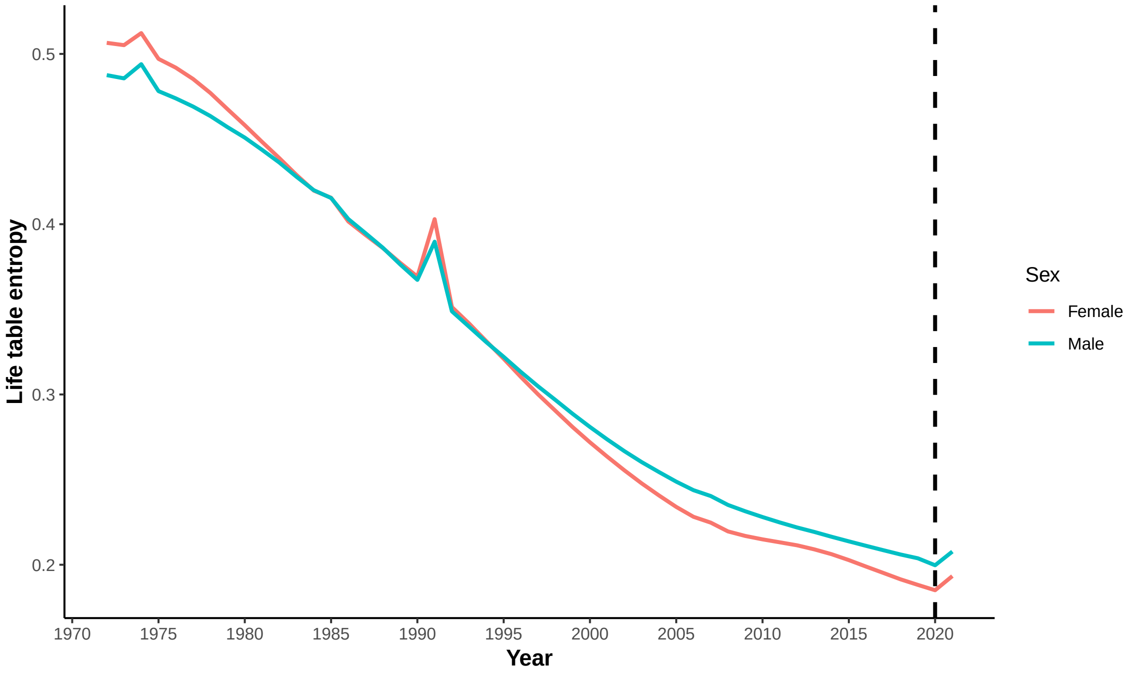

Figure S4: Trend of life table entropy in Bangladesh for both sexes (1972:2021).
